# Effect of Manual Therapy Compared to Ibuprofen on Primary Dysmenorrhea in Young Women—Concentration Assessment of C-Reactive Protein, Vascular Endothelial Growth Factor, Prostaglandins and Sex Hormones

**DOI:** 10.3390/jcm11102686

**Published:** 2022-05-10

**Authors:** Zofia Barcikowska, Magdalena Emilia Grzybowska, Piotr Wąż, Marta Jaskulak, Monika Kurpas, Maksymilian Sotomski, Małgorzata Starzec-Proserpio, Elżbieta Rajkowska-Labon, Rita Hansdorfer-Korzon, Katarzyna Zorena

**Affiliations:** 1 Department of Immunobiology and Environment Microbiology, Medical University of Gdańsk, Dębinki 7, 80-211 Gdańsk, Poland; marta.jaskulak@gumed.edu.pl (M.J.); monika.kurpas@gumed.edu.pl (M.K.); maksymilian.sotomski@gumed.edu.pl (M.S.); kzorena@gumed.edu.pl (K.Z.); 2 Department of Gynecology, Obstetrics and Neonatology, Medical University of Gdańsk, Smoluchowskiego 17, 80-214 Gdańsk, Poland; mlgrzybowska@wp.pl; 3 Department of Nuclear Medicine, Medical University of Gdańsk, Tuwima 15, 80-210 Gdańsk, Poland; piotr.waz@gumed.edu.pl; 4 Department of Midwifery, Centre of Postgraduate Medical Education, Żelazna 90 Str., 01-004 Warsaw, Poland; m.starzec@outlook.com; 5 Department of Physical Therapy, Medical University of Gdańsk, Dębinki 7, 80-211 Gdańsk, Poland; erlabon@gumed.edu.pl (E.R.-L.); rita.hansdorfer-korzon@gumed.edu.pl (R.H.-K.)

**Keywords:** young women, primary dysmenorrhea, manual therapy, ibuprofen, CRP, VEGF, prostaglandins, progesterone, estradiol, physiotherapy

## Abstract

Background: The study aimed to assess if manual therapy, compared to ibuprofen, impacts the concentration of inflammatory factors, sex hormones, and dysmenorrhea in young women Methods: Thirty-five women, clinically diagnosed with dysmenorrhea, were included in the study. They were divided into group A—manual therapy (*n* = 20) and group B—ibuprofen therapy (*n* = 15). Inflammatory factors such as vascular endothelial growth factor (VEGF), C-reactive protein (CRP), prostaglandin F2α (PGF_2α_), E2 (PGE2) and sex hormones levels were measured. Dysmenorrhea assessed with the numerical pain rating scale (NPRS), myofascial trigger points, and muscle flexibility were examined before and after the interventions. Results: The difference in the level of 17-β-estradiol after manual and ibuprofen therapy was significant, as compared to baseline (*p* = 0.036). Progesterone levels decreased in group A (*p* = 0.002) and B (*p* = 0.028). The level of CRP was negatively correlated with sex hormones. Decrease in dysmenorrhea was significant in both groups (group A *p* = 0.016, group B *p* = 0.028). Non-significant differences were reported in prostaglandins, VEGF and CRP levels, in both groups. Conclusions: There were no significant differences in CRP, prostaglandins and VEGF factors after manual or ibuprofen therapy. It has been shown that both manual therapy and ibuprofen can decrease progesterone levels. Manual therapy had a similar effect on the severity of dysmenorrhea as ibuprofen, but after manual therapy, unlike after ibuprofen, less muscles with dysfunction were detected in patients with primary dysmenorrhea.

## 1. Introduction

Dysmenorrhea is defined as pain occurring in the lower abdomen, thighs or back during menstruation and is the most common gynecological problem encountered by women in their reproductive age [1,2,3]. According to the available data, dysmenorrhea can affect up to 97% of women. For young women aged 17–24 years, the reported rate is between 67% and 90% [4,5]. The survey conducted by our team showed that, among Polish respondents, as much as 94% of women have dysmenorrhea [6]. Dysmenorrhea could cause absence from work or school, with an estimated 600 million h missed annually in the United States alone [1,4,7,8].

According to the International Classification of Diseases 10 (ICD-10) dysmenorrhea can be classified as “primary” or “secondary” [9]. Primary dysmenorrhea is defined as the presence of pain during menstruation without any pathological disorders in the pelvic area. When menstrual pain is associated with conditions such as endometriosis, pelvic inflammatory disease, leiomyomas and interstitial cystitis, it is classified as secondary dysmenorrhea [1].

Menstruation is associated with the occurrence of inflammation [10], which is regulated by complex interactions between hormonal, vascular and immune complexes [11]. The decrease in progesterone concentration at the end of the menstrual cycle results in an increased synthesis of prostaglandin E_2_ (PGE_2_) and F_2__α_ (PGF_2__α_). Furthermore, there is an inflow of inflammatory chemokines, cytokines and leukocytes. Progesterone withdrawal induces the expression of matrix metalloproteinases in the endometrium, which dissolve the extracellular matrix and trigger the sloughing of the endometrium [11]. Maybin et al., suggests that the progesterone decrease could also have an impact on tissue repair processes [12].

PGE_2_ is a key inflammatory state and pain mediator. It widens blood vessels, relaxes associated smooth muscles, and inhibits noradrenaline secretion in the sympathetic nerve endings [13]. PGF_2__α_ is a strong smooth muscle stimulator [13,14]. During menstruation, PGF_2__α_ induces contraction of the uterine spiral arteries, which leads to ischemia and hypoxia, resulting in menstrual pain [13,15,16]. As early as 1978, Lundstrom and Green demonstrated a statistically significant difference in serum PGF_2__α_ levels between women experiencing dysmenorrhea and those not affected [13,17]. PGF_2__α_-induced hypoxia plays an important role in the endometrial regeneration mechanism by stimulating the expression of angiogenic factors, such as VEGF [18]. VEGF plays an important role during physiological, as well as pathological, angiogenesis and is involved in neo-angiogenesis processes in the postmenstrual endometrium [12]. Furthermore, VEGF can increase blood vessel permeability during menstruation [10].

In has been shown that CRP concentration increases in women during menstruation, compared to other phases of the menstrual cycle [19]. CRP is a clinically recognized acute phase protein, which, in healthy individuals, should not exceed 3 mg/L [15]. Although it has been shown that the CRP increase during menstruation is not hormone-related [19], a correlation was found between the increase in progesterone and CRP during the luteal phase of the menstrual cycle [20].

Along with progesterone, estradiol is the second major hormone playing a vital role in the menstrual cycle. Estradiol exhibits a range of anti-inflammatory actions, such as stimulating nitric oxide production, scavenging free radicals and promoting cell survival [20]. In the first days of the menstrual cycle, during menstruation, increased estradiol secretion reduces bleeding and thickens the endometrium [21].

The treatment of dysmenorrhea consists of a pharmacological and non-pharmacological approach. The different treatment options for dysmenorrhea have been described in detail in our previous publication [15]. The most commonly used methods to treat dysmenorrhea, with proven efficacy, are pharmacological treatments. The first line of treatment is most often based on non-steroidal anti-inflammatory drugs (NSAID), such as ibuprofen, inhibiting prostaglandin synthesis. NSAIDs have been shown to be more effective than placebo in relieving symptoms of dysmenorrhea but significantly more likely to cause side effects [7,22]. In addition to pharmacotherapy, another treatment modality is physiotherapy, such as manual therapy [23,24,25,26,27]. Manual therapy is a safe method of treatment, used to treat various conditions such as musculoskeletal disorders and visceral problems [24,28,29,30]. Our preliminary research results indicated that manual therapy might effectively relieve pain in young women with dysmenorrhea [23]. However, biological mechanisms activated by manual therapy in dysmenorrhea have not yet been clearly described, as is the case for the effects of manual therapy on inflammatory factors and hormones. Based on the available data, it is suggested that the analgesic effect in dysmenorrhea may be achieved by stimulation of the vagus nerve, provoking reactions from the parasympathetic nervous system [30,31,32]. As a consequence, vasodilation of the blood vessels within the pelvic organs occurs, increasing oxygen perfusion and thus, reducing pain caused by ischemia [31].

The study aimed to assess if manual therapy, compared to well-known ibuprofen, has an impact on the concentration of inflammation factors and sex hormones in young women’s bloodstreams. The secondary aim was to evaluate the effectiveness of manual therapy versus ibuprofen in the treatment of dysmenorrhea.

## 2. Materials and Methods

### 2.1. Data Collection

**Figure 1:**
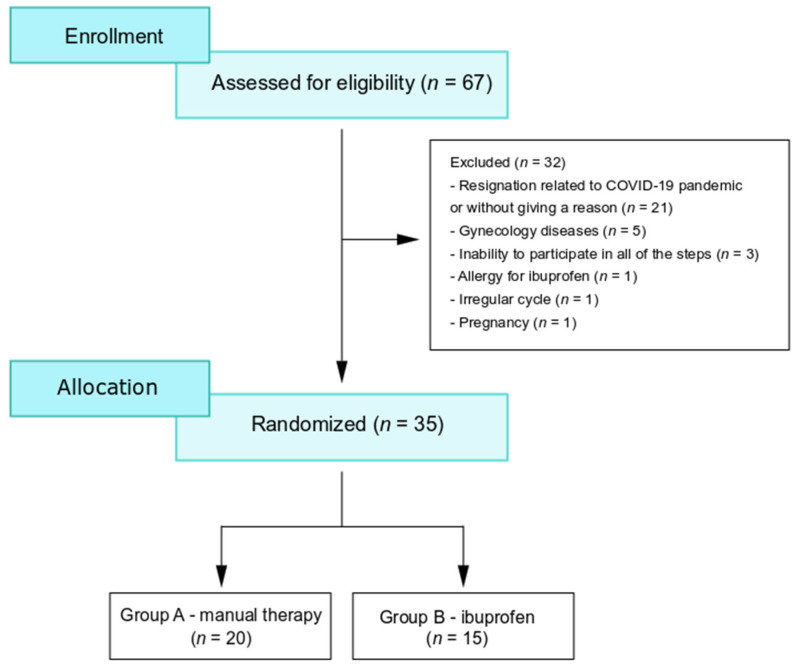
The enrollment of patients with primary dysmenorrhea.

This comparative study was conducted in June 2019–November 2021, at the Medical University of Gdańsk. The subjects were recruited through verbal advertising, social media and the gynecological outpatient clinic. For this study, 67 young women with dysmenorrhea were eligible, of which, 35 women completed the study. Due to their inability to participate in all of the steps, for reasons related to the COVID-19 pandemic, or without giving any reason, 32 women resigned from participation (Figure 1). Based on a simple randomization technique (folded cards with group allocation), patients were randomly divided into two groups: group A treated with manual therapy and group B treated with ibuprofen. The enrollment of patients with primary dysmenorrhea is shown in Figure 1.

Participation in the study was voluntary and informed consent was provided. The study was approved by the Bioethics Committee of the Medical University of Gdansk (No. NKBBN/475/2018). The research was conducted following the principles of the Declaration of Helsinki, as revised in 1996.

### 2.2. Eligibility Criteria

Healthy nulliparous women, aged between 18 and 30, who had regular menstrual cycles and rated dysmenorrhea as five or more points on the numerical pain rating scale (NPRS) [33] were eligible for the study. Patients who met the inclusion criteria underwent a standard gynecological examination, performed by a gynecologist, as well as a transvaginal ultrasound or transabdominal scan for women who had not begun sexual activity. The exclusion criteria were irregular dysmenorrhea, any existing reproductive system diseases, use of hormonal contraception, contraindications to ibuprofen, hip dysplasia, Perthes disease, spinal and abdominal surgery, history of pelvic injuries, regular use of NSAIDs and secondary dysmenorrhea.

### 2.3. Sample Collection

Three blood samples (5 mL each) were taken for assessment of PGE_2_, PGF_2α_, CRP, VEGF, progesterone and estradiol concentrations. They were collected from each subject twice (before and after manual therapy or ibuprofen treatment). The first sample was taken during the first 3 days of the menstrual cycle (between 7:30 and 9:45 a.m.). The second sample was collected during the next menstruation (during the first 3 days) in group A, following manual therapy, and in group B after patients were asked to take ibuprofen. Analgesics were prohibited before the first blood test. The time frames of blood sample collections are depicted in Figure 1.

Levels of 17-β-estradiol and progesterone were analyzed according to the standard procedures of a medical laboratory in Gdańsk, Poland, using the ECLIA electrochemiluminescence method, according to the manufacturer’s instructions.

Blood samples collected to measure the concentration of inflammatory factors were transported to the laboratory within 2 h; each sample was centrifuged at 2500 RPM for 15 min. Obtained blood serum was secured in three Eppendorf tubes (1.5 mL tubes, Eppendorf, Hamburg, Germany), at 500 µL each.

PGE_2_ and VEGF levels were measured by the immuno-enzymatic ELISA method (Quantikine High Sensitivity Human by R&D Systems, Minneapolis, MN, USA) according to the manufacturer’s protocol. PGF_2α_ levels were measured by the immuno-enzymatic ELISA method (Novus Biologicals, Centennial, CO, USA) according to the manufacturer’s protocol. CRP serum levels were measured in the control cohort using the following ELISA kit (Abcam, Cambridge, UK): ab99995 (CRP). Serum samples for CRP measurement were diluted 5000 times in the supplied sample diluent. Absorbance levels were measured on an automated plate reader (ChroMate 4300, Awareness Technology, Inc., Palm City, FL, USA). The reference curves were prepared according to the manufacturer’s recommendations.

### 2.4. Physiotherapy Evaluation

Tender points and flexibility of the following muscles were evaluated: the diaphragm, iliopsoas, thigh abductors, quadratus lumborum, hamstring group, piriformis and tensor fasciae latae. Those muscle groups are indirectly associated with each other; have attachments, or run through the spine area from where the innervation of the uterus comes from. The tenderness was examined according to Simons and Travell [34,35]. The presence of tender points was recorded if the patient reported pain during palpation. The flexibility of the muscles was evaluated by performing a muscle test: measuring the chest circumference during maximum breathing in and out for diaphragm flexibility, a Thomas test for the iliopsoas muscles, passive abduction of the lower limb for the thigh abductor muscles, a lateral bend while standing for the quadratus lumborum muscles, measuring the inferior complement angle at the knee joint for the hamstring group, and a modified Ober’s test for the tensor fasciae latae. All of the examinations were carried out symmetrically on both sides. The presence of muscle dysfunction was defined as the existence of at least one tender point and/or at least one positive muscle test. The physiotherapy evaluation was conducted twice for every patient, before intervention (manual or ibuprofen therapy), and the second time after treatment. The physiotherapy evaluation process was described in detail in our previous paper [23]. All physiotherapy evaluations were performer by the same physiotherapist, who was involved in the manual therapy.

### 2.5. Intervention

#### 2.5.1. Manual Therapy

**Figure 2:**
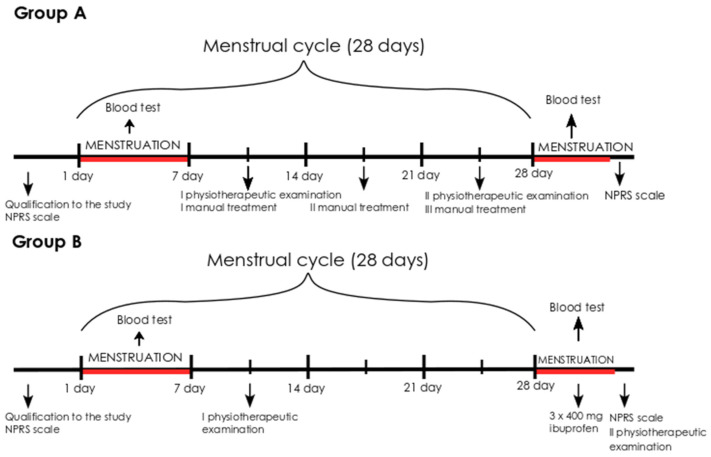
The scheme for carrying out therapy in groups A and B. Modified figure from previous publication [22].

In group A, the subjects underwent manual therapy during one menstrual cycle. The therapy sessions were conducted weekly for 45 min each time. Depending on the length of the menstrual cycle, three or four manual therapy sessions were performed (Figure 2). Manual therapy included diaphragm mobilization [36], normalization of the tone of the pelvic floor muscles (in a supine position with flexed knees, the physiotherapist palpated the pelvic floor muscles on the internal side of the ischial tuberosities; the technique consisted of a little push on the pelvic floor in a cranial direction), post-isometric muscles release [37] and tender point therapy according to Simons and Travell [34,35]. Manual therapy was carried out in every patient, only on the muscles with disorders detected during the physiotherapy examination.

#### 2.5.2. Pharmacological Treatment

Women assigned to receive ibuprofen treatment did not receive manual therapy. Instead, they were advised to take ibuprofen 400 mg, three times per day, during the entire painful episode. The ibuprofen treatment was delivered during the next menstruation following the initial examination.

### 2.6. Statistical Analysis

The results have been generated using the R statistics language [38]. For quantitative variables, basic statistics were calculated, i.e., the mean, median, first quartile (Q1), third quartile (Q3) and standard deviation (σ) values. The normality of the data was assessed using the Shapiro–Wilk test. Numerical values coming from a normally distributed population are characterized by the mean value and by the standard deviation. In the cases for which the result of the Shapiro–Wilk test was statistically significant, the median, and the first and the third quartiles have been used for a description. The differences between the two groups of quantitative variables were tested using the student’s *t*-test or Wilcoxon’s test. The kinds of above-mentioned tests (and additional options) were selected depending on the *p*-value of the Shapiro–Wilk test and the homogeneity of the variance test. The Spearman’s rank correlation coefficients (ρ) and linear models were also used to describe the relationship between pre- and post-treatment values. In the figures showing linear models, the dashed grey line is the set of points for which the pre and post values are identical. Furthermore, all points above the dashed line indicate that the biochemical factor concentrations increased after manual therapy and ibuprofen. Points for group A were marked in red while points for group B were marked in green. In the case of qualitative variables, the frequency of occurrence in the collected research material was determined. For each of the above-mentioned tests, the significance level was set at α = 0.05.

## 3. Results

### 3.1. Characteristics of the Studied Women

The study included 35 women divided into two groups. The age of the study participants was a median 23 (22; 24.5) years and mean 23 ± 2.1 years in group A (*n* = 20), and a median 23 (21.5; 25) years in group B (*n* = 15). The groups did not differ in terms of age, body mass index (BMI), severity of dysmenorrhea and duration of the menstrual cycle (*p* > 0.05). Group B had a significantly longer duration of dysmenorrhea (*p* = 0.046) and menstruation (*p* = 0.016). The basic characteristics of the patients in the study are presented in Table 1.

### 3.2. Examined Factors before and after the Intervention

In Table 2, levels of inflammatory markers and sex hormones are presented.

No significant difference in the initial median and mean PGE_2_ concentrations, in subjects with dysmenorrhea, between groups A and B was observed (*p* = 0.61). After manual therapy, in group A, the median level of PGE_2_ increased from 1044 pg/mL to a mean of 1079.08 pg/mL, but this difference was not significant (*p* = 0.587). In group B, the mean PGE_2_ concentration decreased in a non-significant manner, from 985 pg/mL to 878.9 pg/mL, after ibuprofen intake (*p* = 0.201). Despite changes in PGE_2_ concentrations after manual therapy or ibuprofen intake, the difference between group A and B was not significant (*p* = 0.340) (Table 2).

**Figure 3:**
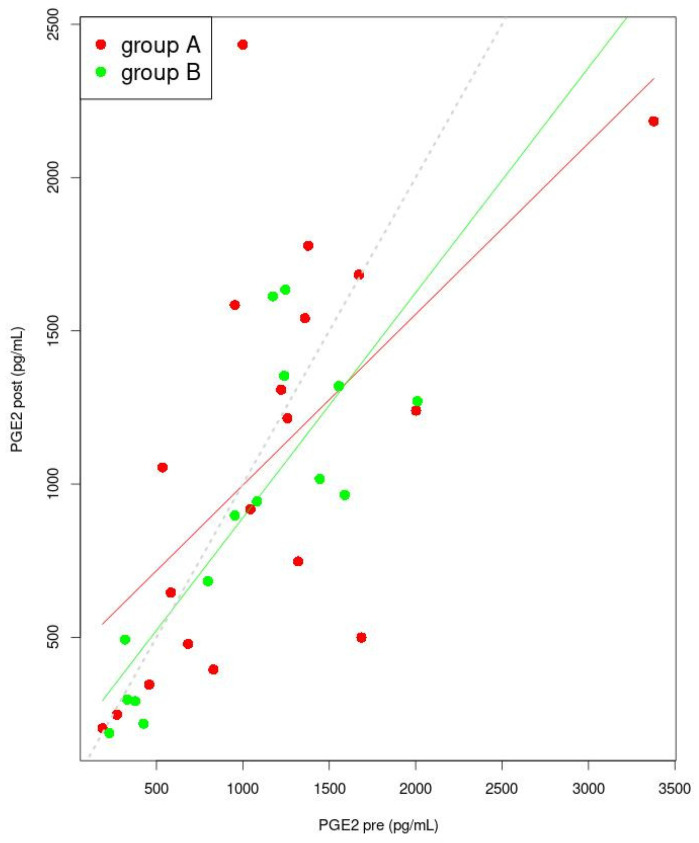
Linear model for PGE_2_ (post dependence on pre). The lines indicate the predicted trend of changes of “pre” and of “post” values (red for group A and green for group B). The dashed grey line is the set of points for which the pre and post values are identical. Points for group A were marked in red, while points for group B were marked in green. Abbreviation: PGE_2_—prostaglandin E_2_.

In Figure 3, the variations in PGE_2_ before treatment (pre) levels, compared to after treatment (post), were presented separately for group A and B, using the general linear model. The designated coefficients used in the general linear model for independent PGE_2_ “pre” are significant (Table 3). Linear models for group A and also group B predict that, when “pre” values increase, for each of the groups there is a point at which “post” PGE_2_ values will start being lower than the “pre” PGE_2_ values. The differences between “pre” and “post” values are increasing faster for group A than for group B.

Before therapy, for median PFG_2α_ concentrations, no significant differences between groups A and B (*p* = 0.791) were shown. After manual therapy, in group A, the median level of PGF_2α_ increased from 2355 pg/mL to a mean of 2689.3 pg/mL but the change was not significant (*p* = 0.409). Among patients from group B, median PGF_2α_ concentration decreased from 2450 to 2030 pg/mL, the difference was not significant (*p* = 0.798). After manual therapy and ibuprofen administration, the difference between groups A and B still were not significant (*p* = 0.945) (Table 2).

Before intervention, the mean concentrations of VEGF between group A and group B were not significantly different (*p* = 0.895). Manual therapy conducted on patients with menstrual pain in group A had a non-significant effect on diminishing VEGF concentration (139.4 vs. 126.6 pg/mL, *p* = 0.141). Ibuprofen intake by women with dysmenorrhea in group B led to a decrease in the mean level of VEGF from 135.2 to 133.8 pg/mL, but the difference was not significant (*p* = 0.754). After manual therapy, no significant difference in VEGF concentration between group A and group B was observed (*p* = 0.938) (Table 2).

**Figure 4:**
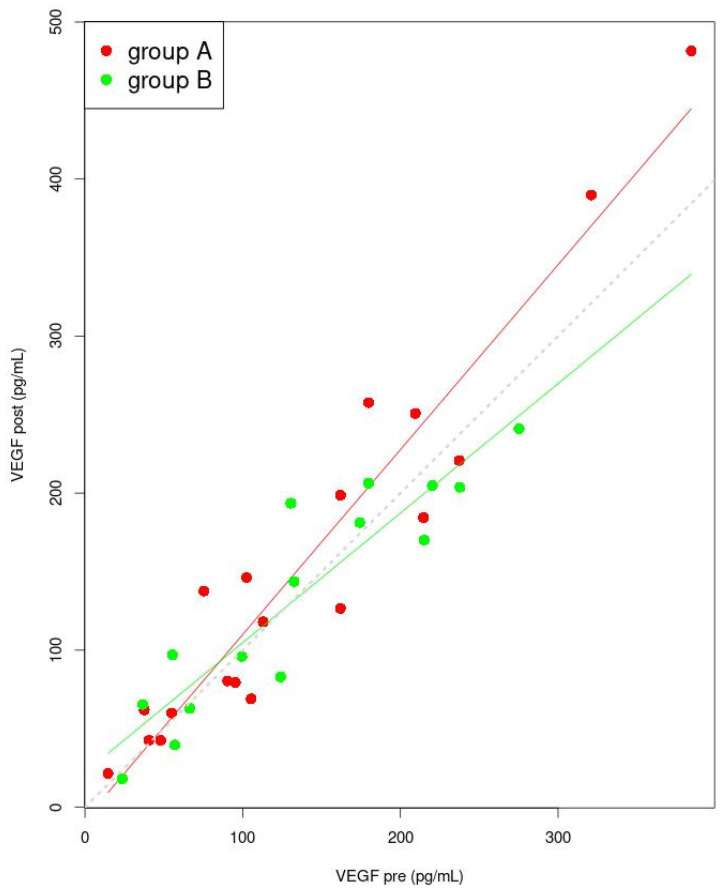
Linear model for VEGF (post dependence on pre). The lines indicate the predicted trend of changes of “pre” and of “post” values (red for group A and green for group B). The dashed grey line is the set of points for which the pre and post values are identical. Points for group A were marked in red, while points for group B were marked in green. Abbreviation: VEGF—vascular endothelial growth factor.

In Figure 4, the general linear model for VEGF concentration for groups A and B pre and post treatment is presented. The designated coefficient used in the general linear model for the independent variable VEGF “pre” is significant (Table 4). The linear model for group A, assumes that when the “pre” value increases, there is a point at which the “post” VEGF values become higher than the “pre” VEGF values. For group B, the linear model predicts that, when the “pre” value increases, there is a point at which the “post” VEGF value becomes lower than the “pre” VEGF.

**Figure 5:**
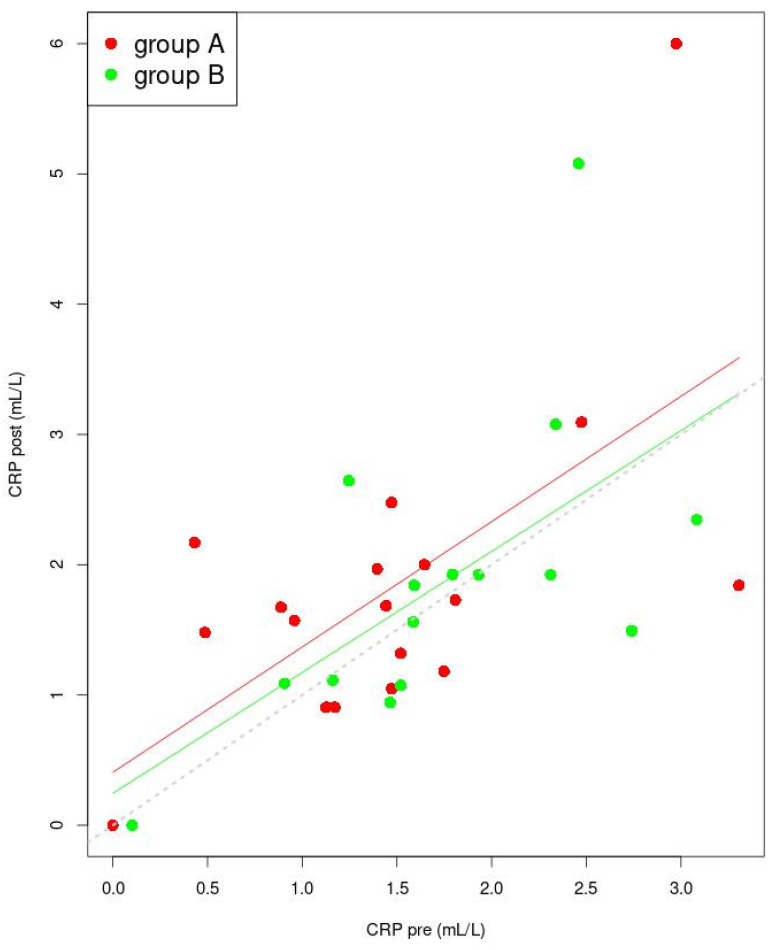
Linear model for CRP (post dependence on pre). The lines indicate the predicted trend of changes of “pre” and of “post” values (red for group A and green for group B). The dashed grey line is the set of points for which the pre and post values are identical. Points for group A were marked in red, while points for group B were marked in green. Abbreviation: CRP—C-reactive protein.

In group A and group B, the mean level of CRP was appropriate for a healthy person (norm to 3 mL/L) [15], and the difference in the mean CRP concentration between the groups was not significant (*p* = 0.613). It was shown that the increase in the mean CRP level in group A, measured after manual therapy (1.386 vs. 1.673 mL/L), was not significant (*p* = 0.097). A non-significant increase in the mean CRP concentration (1.749 vs. 1.869 mL/L) was measured as well, in subjects from group B (*p* = 0.977). After manual therapy or ibuprofen intake, no significant differences between group A and B (*p* = 0.662) were detected (Table 2). The general linear model for the mean CRP concentrations (pre and post) is presented in Figure 5. For each group, there are points for which the CRP “pre” value will become higher than the CRP “post” value. Based on the obtained linear model, it is expected that the CRP concentration will increase, both after manual therapy and ibuprofen administration, in young women with dysmenorrhea. The straight lines for group A and B are almost parallel, which means that the proportional increase in the “post” value relative to the “pre” value, is almost identical in both groups, although the differences between “post” and “pre” in group A are more prominent than in group B. The designated coefficient used in the general linear model for independent variable CRP “pre” is significant (Table 5).

**Figure 6:**
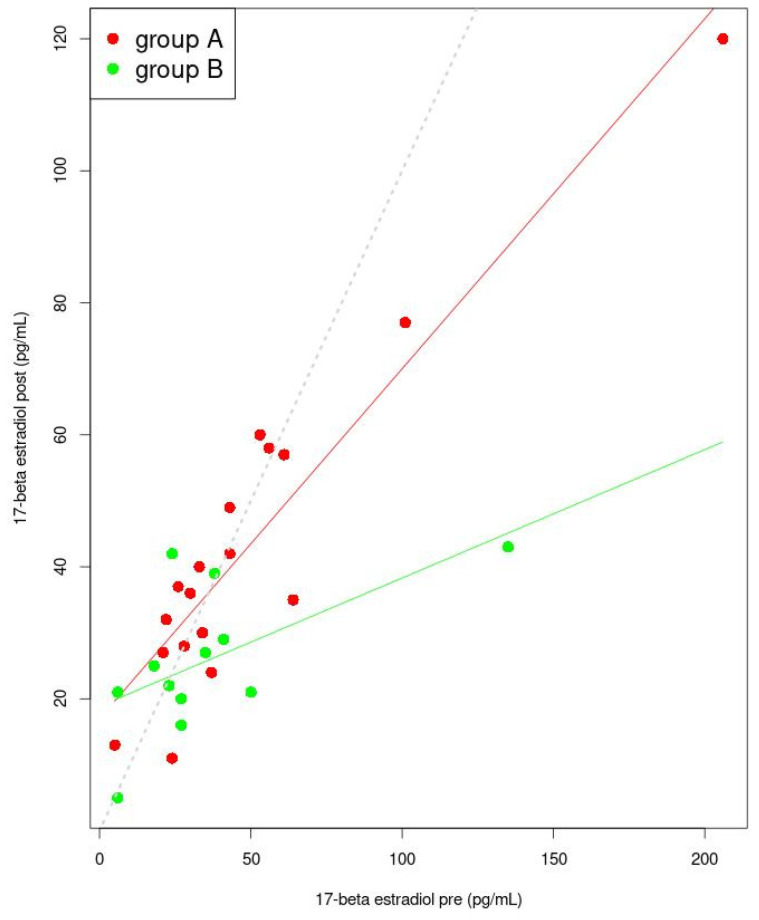
Linear model for 17-β estradiol (post dependence on pre). The lines indicate the predicted trend of changes of “pre” and of “post” values (red for group A and green for group B). The dashed grey line is the set of points for which the pre and post values are identical. Points for group A were marked in red, while points for group B were marked in green.

The differences in the median levels of 17-β estradiol among women from groups A and B before any therapy, were not significant (*p* = 0.229). After conducting manual therapy in group A, an increase in the median 17-β estradiol concentration, from 34 to 36 pg/mL, was measured and the difference was not significant (*p* = 0.776). Among patients from group B, after ibuprofen administration, the median level of 17-β estradiol decreased from 27 to 25 pg/mL and the difference was not significant (*p* = 0.307). Significant differences in the median 17-β estradiol concentration in group A and the mean level in group B, were found after manual therapy compared to ibuprofen intake (*p* = 0.036) (Table 2). In Figure 6, a general linear model for variations in the mean and median 17-beta estradiol levels (pre vs. post) in both groups is presented. Linear models for group A, and also group B, predict that with the increase in the “pre” value for each of the groups there is a point at which the 17-β estradiol “post” value will become smaller than the 17-β estradiol “pre” value. Differences between the “pre” and “post” values increase faster in group B than in group A. The designated coefficient used in the general linear model for independent 17-β estradiol “pre” are significant (Table 6).

At the baseline, the median progesterone concentration in group A was 0.415 pg/mL and 0.35 pg/mL in group B, and the difference was not significant (0.376). After manual therapy for group A patients, a significant (*p* = 0.015) decrease in progesterone levels, to a median of 0.25 pg/mL, was measured. A lower mean progesterone concentration was also measured in group B after ibuprofen administration, at 0.25 pg/mL, and the measured variation was significant (*p* = 0.03). No significant difference was found (*p* = 0.302) in progesterone levels after manual therapy or ibuprofen treatment (Table 2).

### 3.3. Severity of Dysmenorrhea

Based on the NPRS scale, before treatment, both in group A and group B, patients with dysmenorrhea evaluated their median pain on an eleven-point scale (*p* = 0.484). In group A, after manual therapy, the pain rating greatly diminished, from a median of 8, to a mean of 4.9 points on the NPRS scale, the difference was significant (*p* < 0.0001). Among women from group B, after ibuprofen administration, their pain decreased to a median of 3.9 points on the NPRS scale, and the difference was significant (*p* = 0.002). What is more, the changes in severity of dysmenorrhea were clinically significant. The groups did not differ in their pain relief after manual or ibuprofen treatment (*p* = 0.265) (Table 7).

### 3.4. Muscle Dysfunctions

The number of muscles with dysfunction is presented in Table 8. Before treatment, in group A and group B, the median number of muscles showing dysfunction was twelve (*p* = 0.641). After manual therapy, in patients from group A, the number of muscles with dysfunction significantly decreased to a mean of 8.75 (*p* = 0.0005). While in group B, there were no changes in muscle disorders before and after treatment (*p* = 0.112). The difference detected in the number of muscles with dysfunction after manual therapy or ibuprofen intake between group A and B was significant (*p* = 0.001) (Table 8).

### 3.5. Correlation between Sex Hormones and Inflammatory Factor Concentrations

**Figure 7:**
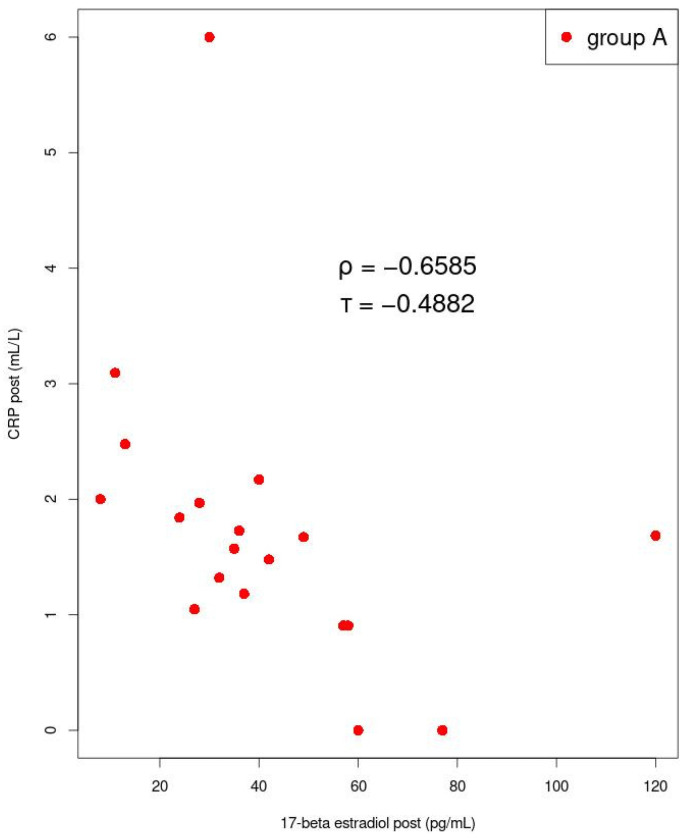
Correlation between 17-beta estradiol and CRP. Kendall’s tau (τ) values and Spearman’s rank correlation rho (ρ) are shown. The determined values of the coefficients are statistically significant.

**Figure 8:**
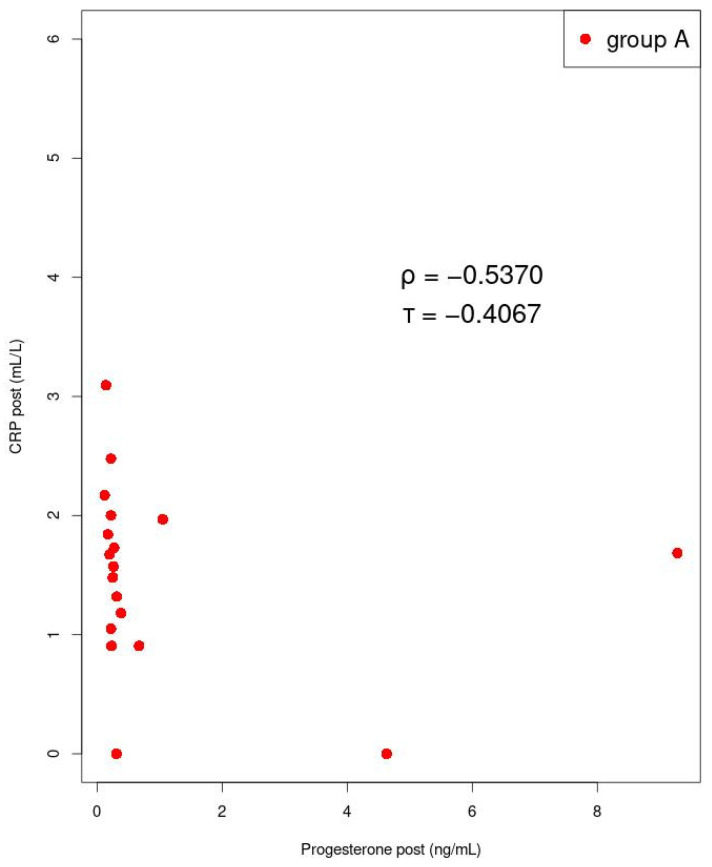
Correlation between progesterone and CRP. Kendall’s tau (τ) values and Spearman’s rank correlation rho (ρ) are shown. The determined values of the coefficients are statistically significant.

Among women with dysmenorrhea in group A after manual therapy, we found a strong negative correlation between mean 17-β estradiol and CRP levels (*p* = 0.002; ρ = −0.659) (Figure 7). A strong negative correlation was also found between mean CRP and progesterone concentrations (*p* = 0.022; ρ = −0.537), in group A, after manual therapy (Figure 8). In group B, during ibuprofen administration, no significant correlations were found.

No statistical correlation between the levels of sex hormones, prostaglandin E_2_, F_2α_ and VEGF was observed.

## 4. Discussion

Our study showed no significant tendency of variations in concentrations of prostaglandins E_2_ and F_2α_, CRP or VEGF after manual therapy or ibuprofen administration in women with primary dysmenorrhea. We showed significant differences in progesterone and 17-β estradiol concentrations after manual therapy and ibuprofen intake. The efficiency of manual therapy, as well as ibuprofen, in relieving dysmenorrhea was proved. To this day, the authors of the publication did not observe any patient deterioration related to an increase in pain after manual therapy. Based on the known literature, it seems that our study is the first to evaluate the impact of manual therapy on progesterone, estradiol and CRP levels in young women with dysmenorrhea.

We compared the impact of manual therapy to ibuprofen administration on PGE_2_ and PGF_2α_ levels. This study detected lower, but not significantly lower PGE2 levels after manual therapy compared to ibuprofen treatment in patients with primary dysmenorrhea. In the available literature, the impact of manual therapy on PGE_2_ was not yet studied. It is known that during menstruation, PGE_2_ has a vasodilatation effect and can inhibit uterus contractility [39,40]. Furthermore, during the whole menstrual cycle, through pro-inflammatory cytokine activity, PGE_2_ impacts the decidualization process and participates in the endometrium repair process [41]. The decrease in PGE_2_ levels in group B, after ibuprofen intake, is compatible with the action mechanism of NSAIDs, as well as with others research [42].

The second analyzed prostaglandin in our study was PGF_2α_. In women from group A, there was a non-significant increase in PGF_2α_ levels after manual therapy, while there was a decrease in PGF_2α_ levels in patients from group B during ibuprofen administration. Although the level of PGF_2α_ increased in women after manual therapy, analgesic effects comparable to ibuprofen were obtained.

The only studies which evaluated the impact of manual therapy on PGF_2α_ metabolites were done by Kokjohn et al. [43] Their results, although indicative of an analgesic effect, showed a decrease in PGF_2α_ metabolites after manual therapy [43]. The difference in influence on PGF_2α_ levels between the two studies could be explained by different times at which manual therapy was applied. In the study by Kokjohn et al., manual therapy was performed during menstruation, whereas in our study, therapy was carried out between two menstruations. Therefore, we suggest that the discrepancy in results may be related to the time period during which manual therapy was applied and the associated physiological tissue responses [43]. In group B, despite that, Dawood et. al. [44] demonstrated a lower concentration of PGF_2α_ in menstrual fluid after ibuprofen intake and we did not detect a significant decrease in PGF_2α_ concentration.

In women from group A, there was a non-significant increase in PGF_2α_ levels after manual therapy, while a decrease in PGF_2α_ levels in patients from group B during ibuprofen administration. In the available literature, only one study that described the impact of massages in rats on VEGF concentration was found [45]. Andrzejewski et al. showed an increase in VEGF-A expression in massaged groups of rats [45]. In studies regarding the influence of NSAIDs on VEGF, it was demonstrated that NSAIDs could block the suppression of VEGF [46,47], which is in line with our research. Based on the latest reports from Jiang et al. [48], who showed higher HIF-1α and VEGF concentrations in rats with active trigger points compared to control rats, we suggest that the tendency to lower VEGF after manual therapy, measured in our patients, might indicate a lower hypoxia occurrence [11].

After manual therapy and ibuprofen administration, we observed a non-significant increase in CRP concentration. Despite a lack of significance between group A and group B before and after therapy, we demonstrated a correlation between sex hormones and CRP in women after manual therapy. We showed a strong negative correlation between 17-β estradiol and CRP concentrations. We have not found other studies estimating the effect of manual therapy on CRP concentrations. The demonstrated correlations between CRP and sex hormone concentrations are compatible with Clancy et al. [49], who showed that higher CRP would be correlated with lower ovarian hormone concentrations. The negative correlation between 17-β estradiol and CRP was also demonstrated by Wander et al., who measured CRP variability during the whole menstrual cycle [19]. Our results, concerning the relationship between progesterone and CRP levels are divergent from the results presented by Wander et al., who measured a simultaneous increase in progesterone and CRP concentrations.

To our knowledge, we were as the first to evaluate the effects of manual therapy on sex hormone levels. No significant differences were detected in the concentrations of 17-β estradiol before manual or ibuprofen therapy. In women from group A, after manual therapy, the 17-β estradiol concentration increased non-significantly whereas, it decreased in women from group B during ibuprofen intake; the difference between groups was significant. The available literature reports that acupuncture administered to perimenopausal women led to higher estradiol levels, which is consistent with the results of our study [50]. A higher estradiol concentration in women after transcutaneous electrical nerve stimulation (TENS) therapy was shown by Ajeena et. al. [51]. TENS treatments were carried out in areas related to the segments of the uterine sympathetic outflow. The authors suggest that the variation in estradiol can occur through increased endorphin secretion that causes gonadotropin secretion. Moreover, one of the study has shown that estradiol concentration is correlated to pain occurrence [52]. In our study, we demonstrated a significant decrease in progesterone concentration after manual therapy, as well as after ibuprofen intake. The effect of manual therapy was similar to ibuprofen. Despite different therapeutic techniques, a similar impact on progesterone levels was shown by Kannan et al. [53]. Kannan et al. [53] assessed the impact of physical activity on progesterone concentrations among women with dysmenorrhea. Sherif et al. reported that, other than ibuprofen, NSAIDs such as naproxen sodium and diclofenac could decrease progesterone levels, which is in line with our results [54]. The diminution of progesterone excretion during the menstrual cycle not only initiates endometrium disintegration but can be a key component of the repair process. The hypoxic and inflammatory environment present during menstruation can, by itself, be responsible for stimulating the expression of endometrial repair factors, which helps to stop bleeding [11].

In group A, after post-isometric relaxation techniques and tender point therapy, we detected less muscles with dysfunction. A similar effect was not observed with ibuprofen use. The improvement of muscle condition demonstrated in this manuscript, proves the results of our previous pilot study [23]. In the available literature, no other studies have analyzed the use of post-isometric relaxation techniques and the therapy of tender points for women with dysmenorrhea. Changes in muscle conditions after manual therapy, in relation to a 17-β estradiol decrease, are compliant with previous results showing that lower estradiol concentrations are correlated to pain occurrence [52].

It has been detected that the analgesic effect of manual therapy was similar to that of ibuprofen. Our results are in concordance with a prior study published by Barassi [55], which showed that neuromuscular manual therapy has a similar effect on dysmenorrhea to ibuprofen or naproxen [55]. Similarly to our studies, it has been shown that other forms of manual therapy such as osteopathy [56,57] and spinal manipulation [58] can relieve dysmenorrhea symptoms.

Significant differences were detected in sex hormone levels. The differences in CRP, VEGF and prostaglandin levels were not significant when comparing before and after manual or ibuprofen treatment. The effect of manual therapy in reducing menstrual pain is described in the literature [55,56,59]; however the action mechanism of manual therapy on sex hormone concentrations is unknown and difficult to describe. Based on our own research [23] and the available literature [31,56,59], we suggest that it may derive from nervous system stimulation, through sex hormones secretion and inflammatory factors modulation. It is known that musculoskeletal disorder occurrence can have an impact on the viscera and autonomic nervous system functions. Kim et al. [60] demonstrated that posture defects such as scoliosis and increased lumbar lordosis led to muscular imbalances and predisposed to dysmenorrhea occurrence. In our research on women with dysmenorrhea, we also detected the presence of muscle dysfunctions and subsequent improvement after manual therapy. In our opinion, the mechanism explaining the analgesic effects of manual therapy can be increased in muscular flexibility. Tissue elasticity improvement entails a better nerve conductivity, diminution of tissue hypoxia and better internal organ position and, as a consequence, their better functioning [23]. Secondly, manual therapy used for pain soothing induces a reaction from the parasympathetic system [31]. It was shown that manual therapy techniques such as spinal manipulations can impact β-endorphin secretion, which in turn, through gonadotropins, can have an effect on estradiol, whose higher concentration has anti-nociceptive effects [52,53,61,62]. In our opinion, manual therapy positively supports natural physiological processes happening in the organism during menstruation. Although linked to an inflammatory state, menstruation is a natural and cyclical process in women. Cyclical endometrium exfoliation leads simultaneously to a cyclical regeneration of itself.

Our study provides additional evidence proving the relevance of adding manual therapy into the dysmenorrhea management process.

It seems that including physiotherapy as an additional method for treating patients with dysmenorrhea, aimed at decreasing pain, is an innovative, non-pharmacological and effective solution. Finally, it is essential to note that physiotherapy procedures used in manual therapy are non-invasive, thus, their implementation is safe and the risk associated with manual therapy is lower than for ibuprofen or other pharmacotherapies.

## 5. Study Limitations

Our study had some limitations. It was conducted on a small group of patients, but this can be associated with the very specific inclusion and exclusion criteria (e.g., women who could not take hormonal contraceptives). Secondly, manual therapy and physiotherapy examinations were conducted by the same physiotherapist who was aware of the group allocation, which could affect the reported results. Additionally, patients were fully aware regarding their received treatment. This could have an impact on the perception of dysmenorrhea, although evaluation of dysmenorrhea was our secondary objective. It is less likely that a lack of blinding had an impact on blood parameters.

## 6. Conclusions

In the presented study, no significant impact on prostaglandins, VEGF and CRP, after manual or ibuprofen therapy, was detected. Manual therapy can decrease progesterone concentrations to a similar extent as ibuprofen. Sex hormone levels were negatively correlated to CRP concentrations. Manual therapy and ibuprofen had a comparable impact on the severity of dysmenorrhea in young women. However, after manual therapy, unlike after ibuprofen, less muscles with dysfunctions were detected in patients with primary dysmenorrhea. The limitations of the study are the lack of blinding, as well as group allocation and physiotherapy being conducted by the same physiotherapist.

## Figures and Tables

**Table 1:** Baseline characteristic of women with primary dysmenorrhea.

	All Patients (*n* = 35)	Group A Manual Therapy (*n* = 20)	Group B Ibuprofen Therapy (*n* = 15)	*p*
Age (years)	23 (22; 24.5)	23.3 ± 2.1	23 (21.5; 25)	0.978 ^a^
Body Mass Index (kg/m^2^)	21.7 ± 2.8	21.9 ± 2.6	21.5 ± 3.2	0.660 ^b^
Severity of dysmenorrhea in NPRS	8 (7; 8)	8 (7; 8)	8 (7; 9)	0.484 ^a^
Duration of menstrual cycle (days)	28 (28; 30.5)	28 (28; 29)	29.5 ± 2.6	0.413 ^a^
Duration of menstruation (days)	6 (5; 6)	5 (5; 6)	6 (5.5; 6)	0.016 ^a^
Duration of dysmenorrhea (days)	2 (2; 3)	2 (2; 3)	3 (2; 3.5)	0.046 ^a^

^a^—Wilcoxon rank sum test with continuity correction, ^b^—Student’s *t*-test, Data presented as: median (Q1; Q3), mean ± standard deviation; Abbreviations: NPRS—numeric pain rating scale, Q—quartile.

**Table 2:** Concentrations of prostaglandins, CRP, VEGF and sex hormones pre and post manual or ibuprofen treatment.

		Group A Manual Therapy (*n* = 20)	Group B Ibuprofen Therapy (*n* = 15)	*p*
PGE_2_ (pg/mL)	Pre	1044 (633.4; 1368.8)	985 ± 554	0.614 ^a^
	Post	1079.08 ± 664.89	878.9 ± 499.8	0.340 ^b^
	*p*	0.587 ^c^	0.201 ^c^	
PGF_2α_ (pg/mL)	Pre	(1245.3; 4615)	2450 (1595; 3457.5)	0.791 ^a^
	Post	2689.3 ± 1904	2030 (1482.5; 3317.5)	0.945 ^a^
	*p*	0.409 ^c^	0.798 ^c^	
VEGF (pg/mL)	Pre	139.4 ± 99.6	135.2 ± 79.3	0.895 ^b^
	Post	126.6 (65.5; 209.7)	133.8 ± 71.3	0.938 ^a^
	*p*	0.141 ^c^	0.754 ^c^	
CRP (mL/L)	Pre	1.386 ± 0.879	1.749 ± 0.764	0.215 ^b^
	Post	1.673 (1.115; 1.984)	1.869 ± 1.165	0.662 ^a^
	*p*	0.098 ^c^	0.977 ^c^	
Estradiol-17β (pg/mL)	Pre	34 (25; 54.5)	27 (19.25; 37.75)	0.229 ^a^
	Post	36 (27.5; 53)	25.462 ± 10.76	0.036 ^a^
	*p*	0.776 ^c^	0.307 ^c^	
Progesterone (ng/mL)	Pre	0.415 (0.262; 0.54)	0.35 (0.25; 0.47)	0.376 ^a^
	Post	0.25 (0.18; 0.33)	0.246 ± 0.133	0.302 ^a^
	*p*	0.016 ^c^	0.028 ^c^	

^a^—Wilcoxon rank sum test with continuity correction, ^b^—Student’s *t*-test, ^c^—Wilcoxon signed rank test with continuity correction; Data presented as: median (Q1; Q3), mean ± standard deviation. Abbreviations: PGE_2_—prostaglandin E_2_; PGF—prostaglandin F_2__α_; VEGF—vascular endothelial growth factor; CRP—C-reactive protein; Q—quartile.

**Table 3:** Linear Model for PGE_2_.

	Estimate	Std. Error	*t*	*p*
(Intercept)	438.410	234.764	1.867	0.079
Group A	0.558	0.174	3.212	0.005
Multiple R-squared: 0.378			Adjusted R-squared: 0.341	
(Intercept)	155.505	162.945	0.954	0.357
Group B	0.734	0.1454	5.053	0.0002
Multiple R-squared: 0.662			Adjusted R-squared: 0.636	

**Table 4:** Linear model for VEGF.

	Estimate	Std. Error	*t*	*p*
(Intercept)	−7.827	14.732	−0.531	0.602
Group A	1.177	0.087	13.572	0.0000000002
Multiple R-squared: 0.916			Adjusted R-squared: 0.910	
(Intercept)	22.242	15.449	1.44	1.74
Group B	0.825	0.099	8.30	0.000001
Multiple R-squared: 0.841			Adjusted R-squared: 0.829	

**Table 5:** Linear Model for CRP.

	Estimate	Std. Error	*t*	*p*
(Intercept)	0.406	0.230	0.946	0.357
Group A	0.962	0.264	3.647	0.002
Multiple R-squared: 0.439			Adjusted R-squared: 0.406	
(Intercept)	0.245	0.637	0.385	0.707
Group B	0.928	0.337	2.767	0.016
Multiple R-squared: 0.371			Adjusted R-squared: 0.322	

**Table 6:** Linear Model for 17-β estradiol.

	Estimate	Std. Error	*t* Value	*p*
(Intercept)	16.981	3.473	4.889	0.0002
Group A	0.530	0.053	10.024	0.00000003
Multiple R-squared: 0.863			Adjusted R-squared: 0.854	
(Intercept)	0.0002	4.059	4.648	0.0009
Group B	0.194	0.084	2.315	0.043
Multiple R-squared: 0.349			Adjusted R-squared: 0.284	

**Table 7:** Severity of dysmenorrhea pre and post manual or ibuprofen treatment.

		Group A Manual Therapy (*n* = 20)	Group B Ibuprofen Therapy (*n* = 15)	*p*
Severity of dysmenorrhea	Pre	8 (7; 8)	8 (7; 9)	0.484 ^a^
	Post	4.9 ± 2.4	3.9 ± 2.8	0.265 ^b^
	*p*	0.000 ^c^	0.002 ^c^	

^a^—Wilcoxon rank sum test with continuity correction, ^b^—Student’s *t*-test, ^c^—Wilcoxon signed rank test with continuity correction; Data presented as: median (Q1; Q3), mean ± standard deviation. Abbreviations: Q—quartile.

**Table 8:** Number of muscles with dysfunction pre and post manual or ibuprofen treatment.

		Group A Manual Therapy (*n* = 20)	Group B Ibuprofen Therapy (*n* = 15)	*p*
Number of muscles with dysfunction	Pre	12 (11; 13)	12 (12; 13)	0.641 ^a^
	Post	8.75 ± 2.552	12 (11; 12.5)	0.002 ^a^
	*p*	0.0005 ^a^	0.112 ^a^	

^a^—Wilcoxon rank sum test with continuity correction, Data presented as: median (Q1; Q3), mean ± standard deviation. Abbreviations: Q—quartile.

## Data Availability

The data presented in this study are available on request from the corresponding author.
